# MCT8 expression in human fetal cerebral cortex is reduced in severe intrauterine growth restriction

**DOI:** 10.1530/JOE-13-0400

**Published:** 2014-01

**Authors:** Shiao Y Chan, Laura A Hancox, Azucena Martín-Santos, Laurence S Loubière, Merlin N M Walter, Ana-Maria González, Phillip M Cox, Ann Logan, Christopher J McCabe, Jayne A Franklyn, Mark D Kilby

**Affiliations:** 1 School of Clinical and Experimental Medicine, College of Medical and Dental Sciences, University of BirminghamEdgbaston, Birmingham, B15 2TTUK; 2 Department of PathologyBirmingham Women's NHS Foundation TrustEdgbaston, Birmingham, B15 2TGUK; 3 Fetal Medicine Centre, Birmingham Women's NHS Foundation TrustEdgbaston, Birmingham, B15 2TGUK

**Keywords:** MCT8, human fetus, CNS, intrauterine growth restriction (IUGR)

## Abstract

The importance of the thyroid hormone (TH) transporter, monocarboxylate transporter 8 (MCT8), to human neurodevelopment is highlighted by findings of severe global neurological impairment in subjects with *MCT8* (*SLC16A2*) mutations. Intrauterine growth restriction (IUGR), usually due to uteroplacental failure, is associated with milder neurodevelopmental deficits, which have been partly attributed to dysregulated TH action *in utero* secondary to reduced circulating fetal TH concentrations and decreased cerebral thyroid hormone receptor expression. We postulate that altered MCT8 expression is implicated in this pathophysiology; therefore, in this study, we sought to quantify changes in cortical MCT8 expression with IUGR. First, MCT8 immunohistochemistry was performed on occipital and parietal cerebral cortex sections obtained from appropriately grown for gestational age (AGA) human fetuses between 19 weeks of gestation and term. Secondly, MCT8 immunostaining in the occipital cortex of stillborn IUGR human fetuses at 24–28 weeks of gestation was objectively compared with that in the occipital cortex of gestationally matched AGA fetuses. Fetuses demonstrated widespread MCT8 expression in neurons within the cortical plate and subplate, in the ventricular and subventricular zones, in the epithelium of the choroid plexus and ependyma, and in microvessel wall. When complicated by IUGR, fetuses showed a significant fivefold reduction in the percentage area of cortical plate immunostained for MCT8 compared with AGA fetuses (*P*<0.05), but there was no significant difference in the proportion of subplate microvessels immunostained. Cortical MCT8 expression was negatively correlated with the severity of IUGR indicated by the brain:liver weight ratios (*r*^2^=0.28; *P*<0.05) at post-mortem. Our results support the hypothesis that a reduction in MCT8 expression in the IUGR fetal brain could further compromise TH-dependent brain development.

## Introduction

Intrauterine growth restriction (IUGR) describes the failure of a fetus to attain its genetically determined growth potential, with the most common underlying etiology being uteroplacental failure associated with abnormal placental development. IUGR is often characterized by continued head and brain growth at the expense of other less vital organs resulting in an elevated brain:liver weight ratio postnatally (Cox & Marton 2009). IUGR complicates 5–10% of pregnancies and is associated with increased perinatal mortality (Kady & Gardosi 2004). Survivors demonstrate an increased prevalence of cognitive impairment compared with babies born appropriately grown for gestational age (AGA). They have lower school achievements and intelligence quotient (IQ) scores (Leitner *et al*. 2007), and 5% show neurodevelopmental delay at the age of 9–10 years (Kok *et al*. 1998). Significantly reduced circulating concentrations of free thyroxine (T_4_) and tri-iodothyronine (T_3_) (Kilby *et al*. 1998) and decreased expression of cerebral thyroid hormone receptor (TR) expression (Kilby *et al*. 2000) in growth-restricted human fetuses are postulated to contribute to this neurodevelopmental morbidity. Examination of growth-restricted fetal guinea pigs has shown a compensatory increase in brain deiodinase type 2 (DIO2) expression, which could increase local concentrations of the active thyroid hormone (TH) ligand, T_3_, from T_4_ conversion (Chan *et al*. 2005). In clinical practice, once IUGR is diagnosed antenatally, timely delivery aimed at avoiding *in utero* demise while prolonging gestation as far as possible for fetal maturity is the mainstay of management. Currently, there are no *in utero* therapies to reduce the risk of neurocognitive impairment in IUGR. An increased understanding of how TH-responsive neurodevelopment is altered in IUGR may lead to the development of novel therapies to improve long-term outcome.

Monocarboxylate transporter 8 (MCT8) is a highly specific plasma membrane TH transporter (Friesema *et al*. 2003). Its importance to human CNS development has been highlighted by discoveries of different mutations within the *MCT8* gene (*SLC16A2*) in subjects with a variety of X-linked mental retardation syndromes, characterized by severe psychomotor and cognitive impairment and accompanied by elevated serum free T_3_ concentrations but normal or low free T_4_ concentrations (Dumitrescu *et al*. 2004, Friesema *et al*. 2004, Schwartz *et al*. 2005).

In mice, MCT8 facilitates the entry of TH into the brain parenchyma across the blood brain–barrier (Ceballos *et al*. 2009) and, at a cellular level, the entry of TH into neurons (Trajkovic *et al*. 2007), where MCT8 is responsible for 75% of T_3_ uptake (Wirth *et al*. 2009). In rodents, TH affects cell proliferation and differentiation of neuroblastoma cells (Garcia-Silva *et al*. 2002) and oligodendrocytes (Jones *et al*. 2003), neuronal migration (Lavado-Autric *et al*. 2003, Auso *et al*. 2004), synaptogenesis (Gilbert & Paczkowski 2003), and cerebellar Purkinje cell dendritic outgrowth (Heuer & Mason 2003). T_3_ has a proproliferative effect in human neuronal precursor cells, NT2, but MCT8, independently of T_3_, could repress NT2 proliferation (James *et al*. 2009), suggesting another role for MCT8 apart from TH transport. However, the lack of neurological defects in *Mct8*-knockout mice (Wirth *et al*. 2009) emphasizes the need for studies in humans. From 7 weeks of gestation, the human fetal cerebral cortex is potentially TH responsive, expressing a range of TH transporters including MCT8 (Chan *et al*. 2011) and all the major TR isoforms (nuclear transcription factors that bind to T_3_ to regulate gene transcription), and demonstrates pre-receptor regulation by DIO2 and deiodinase type 3 (DIO3) (which inactivates T_4_ and T_3_) (Chan *et al*. 2002). Fetal neurons are believed to be the main target for TH action in the brain.

We hypothesize that human fetal cortical MCT8 expression is reduced with severe IUGR, which could further compromise neurodevelopment. In this study, we first carried out localization studies for MCT8 expression in the human fetal cerebral cortex from mid-gestation onwards. We then compared cortical MCT8 expression in severe IUGR human fetuses with that in AGA human fetuses that were stillborn.

## Subjects and methods

### Brain samples

This study was approved by the South Birmingham Research Ethics Committee. Written consent for blocks and slides to be used in research and teaching was obtained in all cases. Cases were identified retrospectively from reports of all post-mortems conducted at the Birmingham Women's Hospital over a 3-year period. Only a minority of cases fulfilled our strict inclusion criteria: normal karyotype, no histopathological evidence of intrauterine infection, and limited or no maceration (indicating very short death-to-delivery intervals). Gestational ages were determined by first-trimester ultrasound scan for crown–rump length. Sections of formalin-fixed paraffin-embedded (FFPE) samples were then obtained from the hospital archive of histopathology blocks.

First, sections of the fetal cerebral cortex (occipital and parietal) obtained during the second (19–20 weeks; *n*=3) and third (26–37 weeks; *n*=3) trimesters from AGA fetuses with unexplained intrauterine deaths were examined. Sections of normal adult occipital cortex (one female aged 55 years and one male aged 37 years) sampled at post-mortem and donated to the London Neurodegenerative Diseases Brain Bank (King's College London, Institute of Psychiatry) were obtained for comparison.

Secondly, sections of the occipital cerebral cortex obtained from stillborn human fetuses between 24 and 28 weeks of gestation were obtained and categorized as either IUGR (*n*=7) or AGA (*n*=5) (Table 1). IUGR was defined as having i) a birth weight below the third percentile for gestation, based on customized growth charts, which account for maternal weight, height, parity, ethnicity, gestation, and fetal sex (Gardosi *et al*. 1992) and ii) a brain:liver weight ratio >4 (Cox & Marton 2009). Although we had not prospectively documented the presence of fetal growth restriction prenatally before death, the post-mortem features were highly suggestive of this pathology. IUGR is likely to be significant, as the phenotype was associated with fetal demise.

### Immunohistochemistry

FFPE sections (5 μm) of cortical samples were immunostained for MCT8 using an avidin–biotin peroxidase technique (Vectastain Elite; all reagents from Vector Laboratories (Peterborough, UK) unless otherwise stated) as per the kit instructions as described previously (Chan *et al*. 2011). Briefly, after dewaxing and serial rehydration, the sections were incubated in 10 mM sodium citrate buffer (pH 6.0) in a 95 °C water bath for 10 min. After washing in 50 mM Tris/0.15 M saline (pH 7.5; TBS), the sections were blocked with 10% normal goat serum (Sigma–Aldrich) in diluting buffer (TBS, 0.3% Tween 20, and 2% BSA) for 20 min. Then, consecutive sections were incubated overnight at 4 °C with rabbit anti-MCT8 (4790) (Sigma–Genosys Ltd., Haverhill, UK; Vasilopoulou *et al*. 2010, Chan *et al*. 2011) at a concentration of 1 μg/ml, anti-glial fibrillary acidic protein (GFAP, glia marker; Dako M0761 at 1:120 (Carpinteria, CA, USA)), or anti-CD68 (microglia marker; Dako M0876 at 1:100). The sections were incubated with biotinylated goat anti-rabbit secondary antibody at 1:200 for 30 min followed by incubation in 5% HRP for 5 min and then in the avidin–biotin–peroxidase complex for 30 min. Immunoreactivity was visualized with 3,3′-diaminobenzidine (15 min). All the steps were separated by TBS–Tween washes. Sections used for localization studies were lightly counterstained with Mayer's hematoxylin and mounted in Vectamount. Slides for comparisons of IUGR fetuses with AGA fetuses were mounted with aqueous Vectashield H1000 without counterstaining. The sections were examined using bright-field microscopy using a Zeiss microscope, and images were captured using the AxioVision Software (Oberkochen, Germany). The specificity of this MCT8 antiserum (4790) has been determined previously (Vasilopoulou *et al*. 2010, Chan *et al*. 2011) and confirmed in the studies by pre-incubating the primary antibody with blocking peptide (25 μg/ml) before application to adjacent sections. Negative control immunostaining was also performed for each tissue sample by omitting the primary antibody.

### Quantification of MCT8 immunostaining

Comparisons of IUGR fetuses with AGA fetuses focused on the occipital cerebral cortex, which is involved in visual perception and interpretation. The *in utero* development of this structure is thought to be TH responsive (Zoeller & Rovet 2004) and affected in IUGR (Dowdeswell *et al*. 1995). We quantified i) the percentage area of cortical plate immunostained for MCT8 and ii) the proportion of microvessels stained for MCT8 in the subplate, with the researcher blinded to the experimental grouping.

#### MCT8 staining in the cortical plate

For each fetus, five images (20× magnification) from the MCT8-immunostained section and five corresponding images from the adjacent section processed with the omission of the primary antibody as a negative control were analyzed. An objective measure of the area containing brown pixels corresponding to immunoreactive staining for MCT8 was quantified using the software ImageJ (U. S. National Institutes of Health, Bethesda, MD, USA) as described previously (Ferreira & Rasband 2012). Briefly, bright-field images were converted to grayscale ‘RGB stack’, and the green channel image was used for analysis. A grayscale cutoff point derived from the corresponding negative controls was set as the threshold signifying positive staining and the same threshold applied to the immunostained sections for each fetus. The total area of tissue stained above the threshold was quantified and expressed as a proportion of the total tissue area examined. The area fraction of background noise, as determined by applying the same threshold to the corresponding negative control, was subtracted from the area fraction of tissue stained to give the true proportion of area of tissue staining positively for MCT8. Since the area of staining could also be affected by cell density, the number of cell bodies (nuclei) within a 250×250 pixel field in each quadrant of every image analyzed was counted and averaged to determine the relative cellularity, which was used to correct the area stained for MCT8. The corrected percentage of area stained for each fetus was then expressed relative to the mean of the AGA group, which was assigned an arbitrary value of 1.

#### MCT8 staining in microvessels

MCT8 immunoreactivity in microvessels was assessed in the subplate zone, a layer deep to the cortical plate with a lower density of cells, where it was easily possible to identify all the microvessels in bright field based on morphology at 40× magnification. For each fetus, 20 non-overlapping images of the subplate were taken. The number of immunostained microvessels was counted and calculated as a percentage of all the microvessels present. An average of 40.4±1.9 microvessels was counted per fetus. Non-specific staining of intravascular erythrocytes was disregarded. The percentage of microvessels stained was then expressed relative to the mean of the AGA group, which was assigned an arbitrary value of 1.

### Statistical analyses

Data were analyzed using the SigmaStat Software, v3.1 (San Jose, CA, USA). Demographic data were analyzed using the unpaired Student's *t*-test to compare continuous variables and the Fisher's exact test to compare contingency tables. Quantitative data expressed as relative values were used for analysis using the two-way ANOVA followed by the Holm–Sidak all pairwise multiple comparisons *post hoc* analysis. The quantitative datasets passed the normality and equal variance tests. Spearman's rank correlation test was used to determine significant correlations between the variables. Significance was taken as *P*<0.05.

## Results

### MCT8 immunolocalization within human fetal and adult cerebral cortex

**Figure 1:**
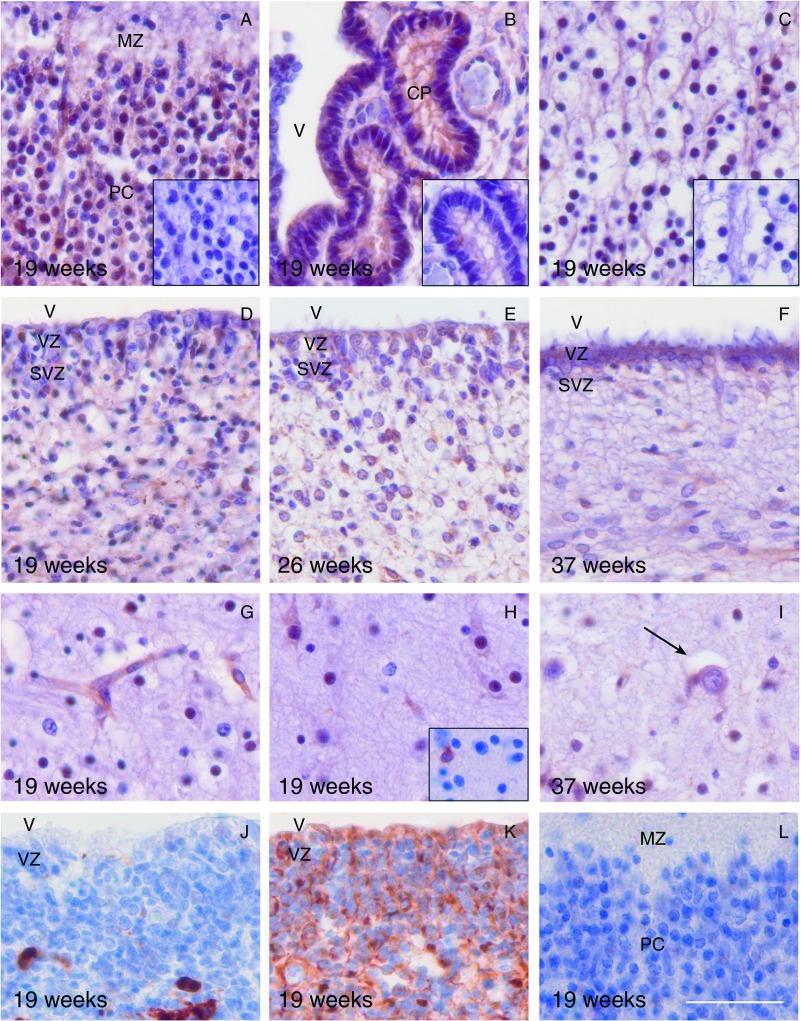
MCT8 immunohistochemistry of cerebral cortex sections obtained from structurally normal fetuses with unexplained intrauterine death. Corresponding negative controls (antibody absorption by the blocking peptide) of adjacent sections are shown in panel inserts in the bottom right corner for (A, B and C). At 19 weeks, MCT8 was located in the cortical plate within the parietal cortex (PC) with less staining in the marginal zone (MZ) (A), in the choroid plexus (CP) (B), and the hippocampus (C). MCT8 immunostaining was also observed in the ependymal cells lining the ventricle (V) and in numerous cells within the ventricular zone (VZ) and subventricular zone (SVZ) at 19 weeks (D), 26 weeks (E), and 37 weeks (F). MCT8 immunostaining was observed in the wall of a microvessel in the subplate at 19 weeks (G) and in neurons in the intermediate zone at 19 weeks (H) and 37 weeks (I; arrow points to a neuron). An adjacent section stained with GFAP is shown in a panel insert in the bottom right corner for (H) showing differences in the morphology of immunostained cells. At 19 weeks, CD68 immunostaining for microglia (J) and GFAP immunostaining for glia (K) in the ventricular and subventricular zones showed a different pattern of staining compared to that of MCT8. There was also a lack of GFAP staining in the cortical plate and marginal zone of the parietal cortex at 19 weeks (L). Magnification bar=50 μm.

The developing human fetal cerebral cortex in mid-gestation is formed by several layers; from superficial to deep, they are the marginal zone, cortical plate, subplate, intermediate zone, subventricular zone, and ventricular zone (lying adjacent to the ventricle; Bystron *et al*. 2008). At 19 weeks of gestation, sections of the parietal and occipital cortex obtained from AGA fetuses demonstrated MCT8 immunostaining in all the layers. Immunostaining was found within the marginal zone, in cortical plate neurons, a proportion of cells in the subplate zone, in hippocampal neurons, epithelial cells of the choroid plexus and ependyma, and in numerous cells in the ventricular and subventricular zones (Fig. 1A, B, C, D and H). A similar distribution of MCT8 immunostaining was observed in sections obtained from AGA fetuses at 26–37 weeks of gestation. However, with advancing gestation and maturity of the cortex, there were fewer cells in the ventricular and subventricular zones and, hence, correspondingly less MCT8 staining in these layers (Fig. 1D, E and F). Most microvessels throughout the areas studied were MCT8 positive (Fig. 1G). The absorption of the antibody with the blocking peptide effectively abolished MCT8 staining, confirming the specificity of staining (Fig. 1A, B and C).

MCT8 immunostaining corresponded with the well-described pattern of neuronal cell distribution within the cerebral cortex, with the greatest staining being observed in the cortical plate, which is dense with neurons. Neuronal localization of MCT8 was also supported by our findings that immunostaining for GFAP and CD68, indicating glia and microglia respectively, in adjacent sections revealed an entirely different pattern of distribution in all the layers of the cortex (Fig. 1J and K) compared with immunostaining for MCT8. Specifically, there was no GFAP (Fig. 1L) or CD68 immunostaining in the cortical plate. In addition, the morphology of cells stained with each antibody was clearly different. Neurons were identified by a round dense nucleus and abundant cytoplasm with dendritic branches, many of which were immunostained for MCT8, in contrast to astrocytes, which had a large irregular nucleus with clear nucleoplasm containing a vesicular chromatin pattern and very small or absent nucleoli showing no MCT8 immunostaining (Fig. 1H and I). Within the subplate only a selected population of neurons were MCT8 positive at every gestational age examined. In the adult occipital cortex, microvessels were immunostained for MCT8, but proportionally fewer neurons were immunostained for MCT8 compared with those in the fetal cortex (data not shown).

### Comparison of MCT8 immunostaining in the occipital cortex of AGA and IUGR fetuses

There were no significant differences between the IUGR and AGA cohorts in terms of gestational age and fetal sex (Table 1). Compared with the AGA group, the IUGR group had significantly lower raw birth weights (*P*<0.05; with all the customized birth weight percentiles being under the third percentile), but the raw brain weights were not significantly different between the two groups, with brain weights being well preserved for gestation even in the IUGR cohort (1.08 relative to the expected mean). However, the relative brain weights (ratio to the expected mean for gestation) in the IUGR group were still lower compared with those in the AGA group (*P*<0.05). The brain:liver weight ratios in the IUGR group were significantly higher compared with those in the AGA group (*P*<0.01). Atrophy of the thymus secondary to chronic stress in IUGR (Cox & Marton 2009) was also evident by the significantly reduced raw thymus weights (*P*<0.01) and thymus weights relative to the expected mean for gestation (*P*<0.001). All these indicate that the IUGR cohort comprised cases at the severe end of the spectrum. Most of the IUGR cases demonstrated features of chronic uteroplacental failure on placental examination (Table 1; ReCoDe C4 and C5), which were absent in the AGA cohort.

**Figure 2:**
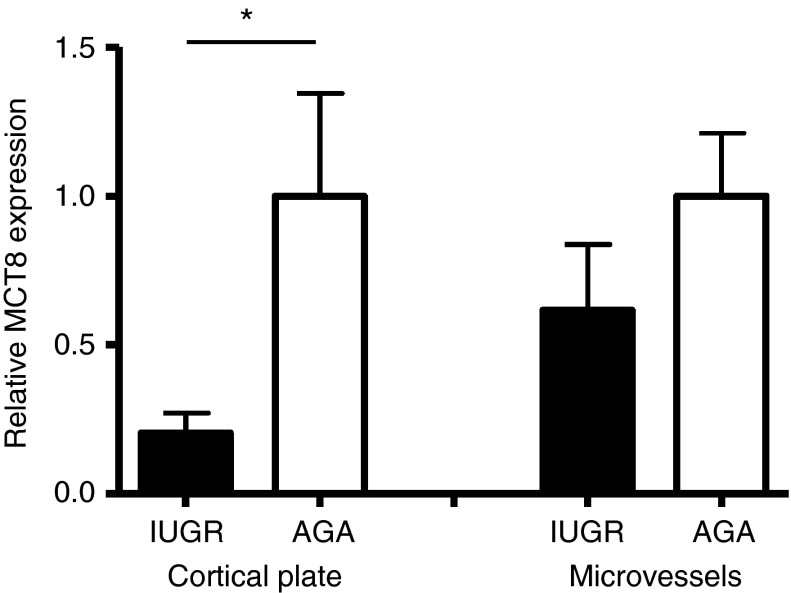
Quantification of MCT8 immunostaining in the occipital cerebral cortex of intrauterine growth-restricted (IUGR) fetuses (*n*=7; black bars) compared with that in the occipital cerebral cortex of appropriately grown for gestational age (AGA) fetuses (*n*=5; white bars). The percentage area of cortical plate and the proportion of microvessels in the subplate immunostained for MCT8 are expressed relative to the mean of the AGA group, which was given an arbitrary value of 1. Columns and error bars represent the mean and s.e.m. Statistically significant difference, *P<0.05.

The overall two-way ANOVA, which analyzed the entire dataset, indicated significantly reduced MCT8 expression in the occipital cortex of IUGR fetuses compared with that in AGA fetuses (*P*<0.05). However, *post hoc* tests indicated that the difference was significant only for cortical plate immunostaining (*P*<0.05; Fig. 2).

**Figure 3:**
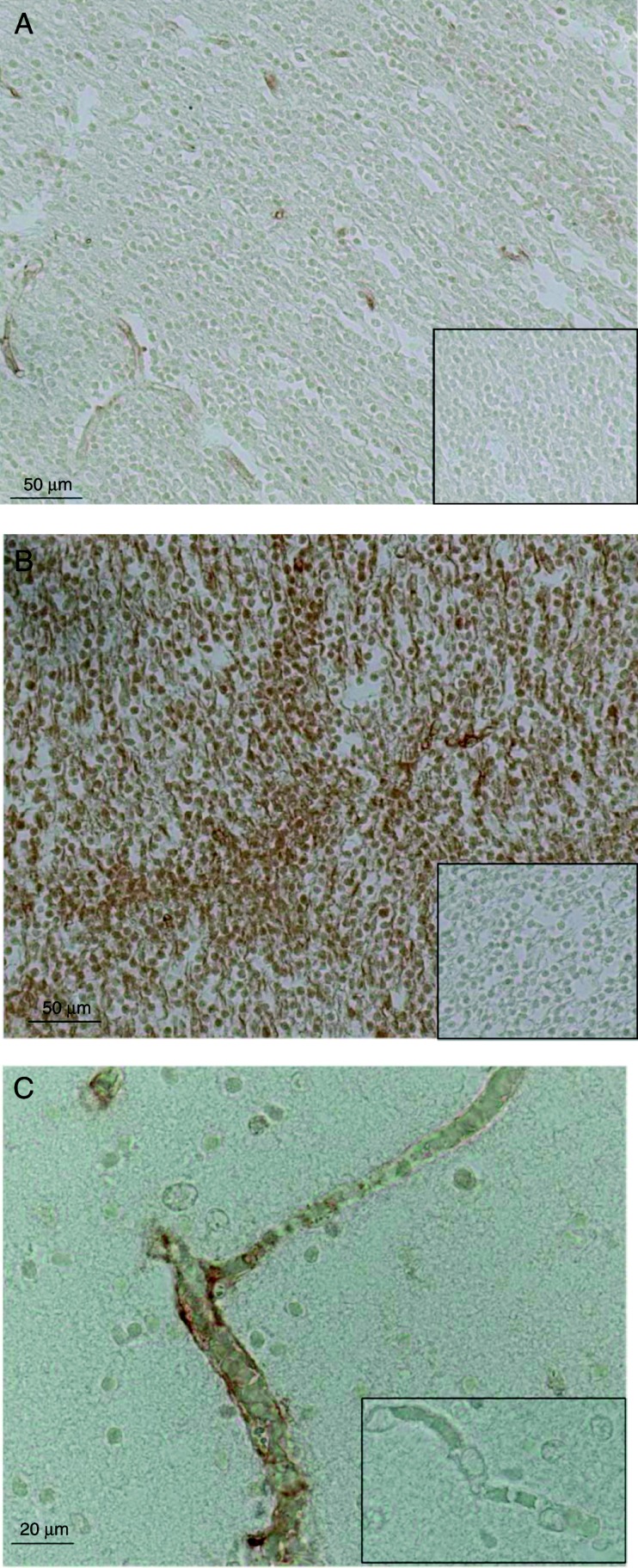
Representative sections showing MCT8 immunostaining of the cortical plate of an intrauterine growth-restricted (IUGR) fetus (A) and an appropriately grown for gestational age (AGA) fetus (B) within the occipital cerebral cortex. Corresponding negative controls (no primary antibody) of adjacent sections are shown in a panel insert in the bottom right corner. An example of a positively MCT8-immunostained microvessel in the subplate (C) compared with a negative one (shown in a panel insert in the bottom right corner) from the same section immunostained for MCT8 is shown.

The mean percentage area of cortical plate immunostaining after correction for relative cell number was 4.7±1.5% (mean±s.e.m.; 0.2±0.07 relative to AGA) in the IUGR group compared with 23.3±8.1% (1±0.3 relative to AGA) in the AGA group (*P*<0.05), which represents approximately a fivefold decrease in MCT8 expression with IUGR (Fig. 2). The cellularity within the cortical plate was not significantly different between the two cohorts (IUGR, 1.3±0.1 and AGA, 1±0.1). General observations of cortical plate images indicated that the decrease in MCT8 staining was confined to morphologically defined neuronal cells, while microvessels seemed to be spared (Fig. 3).

*Post hoc* tests revealed no statistically significant difference in the proportion of microvessels stained for MCT8 in the IUGR samples (27.9±10.0%; 0.6±0.2 relative to AGA) compared with that in the AGA samples (45.2±9.6%; 1±0.2 relative to AGA; Fig. 2).

**Figure 4:**
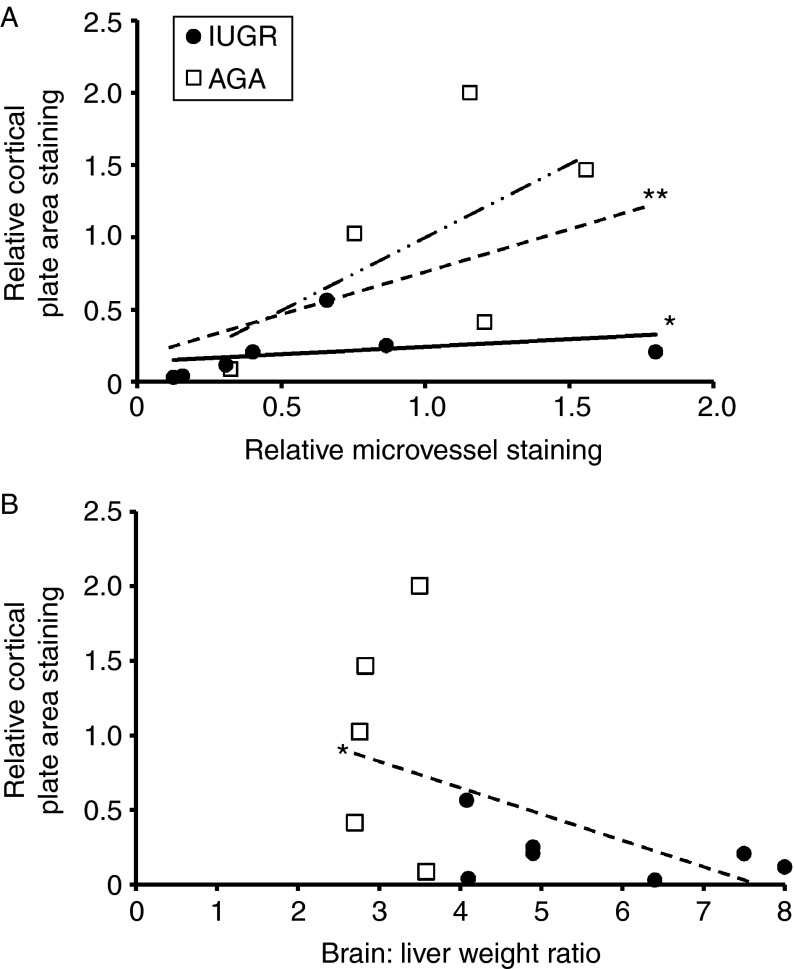
(A) Correlation between the relative cortical plate area immunostained for MCT8 and the relative proportion of microvessels immunostained for MCT8 in IUGR (black dots) and AGA (white squares) fetuses. A significant positive correlation was observed when all the samples were considered together (dashed line) and when only the IUGR samples were considered on their own (straight line), but there was no significant correlation when only among the AGA samples were considered on their own (dashed and dotted line). (B) Correlation between the relative cortical plate area immunostained for MCT8 and the brain:liver weight ratio. A negative correlation was observed when all the samples were considered together. Statistically significant differences are **P<0.01, *P<0.05.

However, there was a significant positive correlation between the area of cortical plate MCT8 immunostaining and the proportion of microvessels stained in the subplate (correlation coefficient=0.71, *r*^2^=0.27; *P*<0.01) when all the samples were analyzed together. The positive correlation remained significant within the IUGR group (correlation coefficient=0.75, *r*^2^=0.12; *P*<0.05; Fig. 4A), but there was no significant correlation within the AGA cohort on its own.

When all the samples were analyzed together, a negative correlation was also observed between the area of cortical plate MCT8 immunostaining and brain:liver weight ratios (correlation coefficient=−0.64, *r*^2^=0.28; *P*<0.05; Fig. 4B). There was no correlation between MCT8 immunostaining in the cortical plate or microvessels with either gestational age or fetal sex.

## Discussion

Changes in TH transporter expression have never been described in the growth-restricted state. This study is the first to demonstrate significantly reduced cortical MCT8 expression within the developing CNS of human fetuses stillborn with severe IUGR. Our results suggest that altered TH transporter activity in cerebral neurons could be a contributory factor to the pathophysiology of neurodevelopmental impairment associated with IUGR.

The strength of this study is the use of human fetal tissue samples, thus eliminating species differences, particularly relevant, as *Mct8*-knockout mice lack the neurological phenotype observed in humans with *MCT8* mutations. However, a limitation is the restriction of the availability of human fetal tissue samples of adequate quality for investigation, hence, the small number of samples in this study.

The localization of MCT8 in developing neurons across the different cortical layers, microvessels, and choroid plexus reported herein is generally consistent with the findings of previously published studies of human fetuses (Roberts *et al*. 2008, Wirth *et al*. 2009) and supports its role in the uptake of TH into the brain parenchyma from the blood and cerebrospinal fluid, as well as into neurons, from early fetal development.

Neurogenesis takes place in the ventricular and subventricular zones with much being completed by 28 weeks of gestation (Bystron *et al*. 2008). MCT8 staining in these neuroprecursor-rich areas at 19–26 weeks suggests its involvement in the regulation of neurogenesis. Indeed, we have previously demonstrated that MCT8 represses the proliferation of the human neuronal precursor cell NT2 in a T_3_-independent manner (James *et al*. 2009); however, MCT8 had no effect on NT2 neurodifferentiation *in vitro* (Chan *et al*. 2011). Post-mitotic neurons migrate away from the proliferative zones and, by 24–28 weeks of gestation, most cortical neurons have settled to form the cortical plate, an area comprising predominantly neuronal cells (Bystron *et al*. 2008).

During normal human fetal cortical development, over 70% of neurons undergo programmed cell death after 32 weeks of gestation (Rabinowicz *et al*. 1996). Magnetic resonance imaging (MRI) assessments of IUGR premature infants at 33–34 weeks have shown reduced cerebral cortical gray matter volume (Tolsa *et al*. 2004), which could be due to reduced cell numbers in the cortical plate (Samuelsen *et al*. 2003). Similar to that observed in our IUGR cohort at 24–28 weeks, that study (Samuelsen *et al*. 2003), however, found no significant differences in cortical cell numbers compared with AGA samples before 27 weeks.

MCT8 promotes cell death in non-proliferative cytotrophoblast cells from human placenta independently of T_3_ (Vasilopoulou *et al*. 2013). It remains speculative whether MCT8 could also have a similar effect on neuronal apoptosis. If so, the downregulation of MCT8 expression in IUGR neurons could be a protective mechanism to limit neuronal apoptosis at the expense of TH transport, of which the latter could be partially compensated for by other TH transporters expressed by neurons, as we and others have previously described in the human fetal cerebral cortex (Wirth *et al*. 2009, Chan *et al*. 2011).

Whether the reduction in cortical cell number in the third trimester is due to reduced neurogenesis, reduced neuronal migration, or increased cell death in IUGR is not known. In rats, abnormal neuronal migration in the fetal CNS in both IUGR (Sasaki *et al*. 2000) and TH deficiency (Auso *et al*. 2004) has been reported. Maternal TH deficiency in rats has also been reported to lead to impaired neurogenesis and diminished neocortical neuronal numbers (Mohan *et al*. 2012). The extent to which these altered cellular processes in IUGR, as well as possibly altered synaptogenesis and dendritic branching, are mediated by diminished TH action secondary to reductions in circulating TH concentrations, MCT8 transport, and TR expression remains the subject of investigation. Other factors such as cerebral hypoxia and prematurity are also likely to contribute to this neuropathology. Whatever the etiologies, alterations in brain neural networks assessed by MRI in IUGR infants have been found to be associated with later neurodevelopmental outcomes (Batalle *et al*. 2012).

Current understanding of the physiological regulation of MCT8 expression is poor. TH status has been shown to influence MCT8 expression in some tissues (Capelo *et al*. 2009) but not in others (Mebis *et al*. 2009). In IUGR, MCT8 expression in the human placenta is upregulated (Vasilopoulou *et al*. 2010) in contrast to that in the fetal cerebral cortex. These tissue-specific effects argue against a general alteration in MCT8 activity being part of the etiology of IUGR, but rather suggest that altered cerebral MCT8 expression is a local adaptive response to the growth-restricted state that is associated with chronic distress, which is supported by our finding that the greater the growth restriction, the lower the MCT8 expression. This is in contrast to the AGA fetuses that presumably suffered from an acute event just before death. The positive correlation between MCT8 expression in the cortical plate and that in microvessels suggests that there may be some common mechanisms regulating MCT8 expression in the CNS.

Future studies should investigate whether there are compensatory alterations in the expression of other TH transporters in neurons and microvessels. Studies could also extend to other regions of the CNS and at different gestational ages to obtain a more comprehensive picture of the effects of IUGR on TH transport and how this could correlate with observed neurological impairments in IUGR survivors.

In conclusion, our results showing perturbed patterns of cortical MCT8 expression support the hypothesis that a reduction in MCT8 expression in the IUGR fetal CNS could be a contributory factor implicated in the long-term neurodevelopmental impairments associated with this condition.

## Figures and Tables

**Table 1:** Characteristics of human stillbirth cases.

**Case**	**Gestation** (weeks+days)	**Sex** (M/F)	**Fetal weight** (g) (customized percentile (%))	**Brain weight** (g)	**Brain weight relative to the expected mean for gestation**	**Brain:liver weight ratio**	**Thymus weight** (g)	**Thymus weight relative to the expected mean for gestation**	**Cause of death** (ReCoDe)
Intrauterine growth restriction (IUGR)									
IUGR1	24+0	F	376 (<1)	76	0.92	4.9	0.2	0.13	Antepartum asphyxia (A8 – cause unknown)
IUGR2	26+0	M	643 (<1)	134	1.28	8.0	0.4	0.2	Antepartum asphyxia (A8, C4)
IUGR3	26+1	F	606 (<1)	117	1.11	4.9	0.4	0.2	Antepartum asphyxia (A8, C4)
IUGR4	26+3	M	592 (<1)	106	1.01	7.5	0.4	0.2	Antepartum asphyxia (A8, C5)
IUGR5	27+1	F	709 (<1)	130	1.10	4.1	1.2	0.52	Antepartum asphyxia (A8, C1, C4)
IUGR6	27+6	F	746 (<1)	135	1.14	6.4	1.4	0.61	Antepartum asphyxia (A8, C5)
IUGR7	28+2	M	906 (<1)	136	1.03	4.1	0.8	0.31	Antepartum asphyxia (A8, B4, C4)
Mean±s.e.m.	26.6±0.5 weeks	M:F, 3:4	654±61	119±8	1.08±0.04	5.7±0.6	0.69±0.17	0.31±0.07	
Appropriately grown for gestational age (AGA)									
AGA1	24+2	M	732 (75)	102	1.23	3.5	1.4	0.93	Intrapartum asphyxia (A7, C1)
AGA2	25+0	F	688 (50)	105	1.12	2.7	2.5	1.39	Antepartum asphyxia (C1)
AGA3	26+2	M	863 (10)	123	1.17	2.8	1.5	0.75	Intrapartum asphyxia (A9, D2)
AGA4	26+6	F	938 (77)	131	1.25	2.8	2.5	1.25	Intrapartum asphyxia (C1)
AGA5	28+1	M	1156 (34)	176	1.49	3.6	3.8	1.65	Antepartum asphyxia (C1)
Mean±s.e.m.	26.1±0.7 weeks	M:F, 3:2	875±83	127±13	1.25±0.06	3.1±0.2	2.34±0.43	1.19±0.16	
*P* value (IUGR vs AGA)	NS	NS	0.05	NS	0.049	0.006	0.003	<0.001	

ReCoDe is a classification system for stillbirths by relevant condition at death (Gardosi *et al.* 2005). A7, intrapartum asphyxia; A8, fetal growth restriction; A9, other fetal factor (in AGA3, it was pulmonary hypoplasia); B4, other umbilical cord (in IUGR7, it was umbilical vein thrombosis); C1, abruption; C4, placental infarction; C5, placental insufficiency; D2, oligohydramnios.
